# Autistic-Like Behavior and Impairment of Serotonin Transporter and AMPA Receptor Trafficking in *N*-Ethylmaleimide Sensitive Factor Gene-Deficient Mice

**DOI:** 10.3389/fgene.2021.748627

**Published:** 2021-10-20

**Authors:** Min-Jue Xie, Keiko Iwata, Yasuyuki Ishikawa, Yuki Nomura, Tomomi Tani, Koshi Murata, Yugo Fukazawa, Hideo Matsuzaki

**Affiliations:** ^1^ Division of Development of Mental Functions, Research Center for Child Mental Development, University of Fukui, Fukui, Japan; ^2^ Life Science Innovation Center, University of Fukui, Fukui, Japan; ^3^ United Graduate School of Child Development, Osaka University, Kanazawa University, Hamamatsu University School of Medicine, Chiba University and University of Fukui, Osaka University, Osaka, Japan; ^4^ Department of Systems Life Engineering, Maebashi Institute of Technology, Maebashi, Japan; ^5^ School of Medicine, Faculty of Medical Sciences, University of Fukui, Fukui, Japan; ^6^ Division of Brain Structures and Function, Department of Morphological and Physiological Sciences, Faculty of Medical Sciences, University of Fukui, Fukui, Japan

**Keywords:** serotonin transporer, N-ethylmaleimide-sensitive factor, autism spectrum disorder, AMPA receptor, behavior

## Abstract

Autism spectrum disorder (ASD), characterized by profound impairment in social interactions and communication skills, is the most common neurodevelopmental disorder. Many studies on the mechanisms underlying the development of ASD have focused on the serotonergic system; however, these studies have failed to completely elucidate the mechanisms. We previously identified *N*-ethylmaleimide-sensitive factor (NSF) as a new serotonin transporter (SERT)-binding protein and described its importance in SERT membrane trafficking and uptake *in vitro*. In the present study, we generated *Nsf* ^*+/-*^ mice and investigated their behavioral, neurotransmitter, and neurophysiological phenotypes *in vivo*. *Nsf* ^*+/-*^ mice exhibited abnormalities in sociability, communication, repetitiveness, and anxiety. Additionally, *Nsf* loss led to a decrease in membrane SERT expression in the raphe and accumulation of glutamate alpha-amino-3-hydroxy-5-methyl-4-isoxazole propionic acid receptors at the synaptic membrane surface in the hippocampal CA1 region. We found that postsynaptic density and long-term depression were impaired in the hippocampal CA1 region of *Nsf* ^*+/-*^ mice. Taken together, these findings demonstrate that NSF plays a role in synaptic plasticity and glutamatergic and serotonergic systems, suggesting a possible mechanism by which the gene is linked to the pathophysiology of autistic behaviors.

## Introduction

Autism spectrum disorder (ASD) is a neurodevelopmental disorder characterized by severe and sustained impairment of social interaction and communication and restricted or stereotyped patterns of behavior and interest. Multiple risk factors, comprising both genetic and environmental factors, are known to be associated with the onset of ASD, indicating the complex etiology of this disorder. Research focusing on neurotransmitters has been conducted, and accumulating evidence suggests that both serotonin (5-HT) and glutamine (Glu) neurotransmitter systems are implicated in the onset and progression of ASD (Eissa et al., 2018).

5-HT signaling facilitates several neural processes, including neurogenesis, cell migration and survival, synaptogenesis, and synaptic plasticity. Previous studies have consistently found elevated serotonin levels in whole blood cells and platelets of patients with autism (Schain, 1961; Hanley et al., 1977; Ciaranello, 1982; Anderson et al., 1987; Cook et al., 1988) and their relatives (Abramson et al., 1989; Cook et al., 1990; Cross et al., 2008). Short-term dietary tryptophan (precursor of 5-HT) depletion has been shown to exacerbate repetitive behavior and elevate anxiety and feelings of unhappiness in adults with autism (McDougle et al., 1996). A single-photon emission computed tomography study revealed that children with autism have reduced serotonin transporter (SERT) binding in the medial frontal cortex, midbrain, and temporal lobe (Makkonen et al., 2008). SERT is an integral plasma membrane glycoprotein that regulates neurotransmission through the reuptake of 5-HT from the synaptic cleft. Importantly, SERT expression, determined using radioligand binding assay results, has been reported to be significantly lower throughout the brain in individuals with autism than in controls (Nakamura et al., 2010). In contrast, *SERT* mRNA expression has not been found to significantly change in brain samples and lymphocytes of patients with ASD (Iwata et al., 2014). These findings suggest that SERT expression at the membrane surface and 5-HT transport capacity are decreased in the brains of ASD patients.

Increased levels of Glu have been found in the blood samples of children and adults with ASD (Moreno et al., 1992; Moreno-Fuenmayor et al., 1996; Aldred et al., 2003; Shinohe et al., 2006; Shimmura et al., 2011; Tirouvanziam et al., 2012). Glu levels in the brain have been assessed *in vivo* using proton magnetic resonance spectroscopy. Several groups have reported significantly increased Glu levels in several brain regions, including the anterior cingulate gyrus (Bejjani et al., 2012; Joshi et al., 2013) and the auditory cortex (Brown et al., 2013). Alpha-amino-3-hydroxy-5-methyl-4-isoxazole propionic acid (AMPA) receptors, which are tetrameric (GluA1–GluA4) and cation-permeable ionotropic glutamate receptors, are expressed throughout the brain (Beneyto and Meador-Woodruff, 2004). Intriguingly, receptor autoradiography results have revealed that the AMPA receptor density is slightly decreased, while post-mortem studies have revealed that *GluA1*-*GluA3* mRNA levels are significantly increased in the brains of ASD individuals (Purcell et al., 2001b). These findings suggest that AMPA receptor expression at the membrane surface and its function are impaired in the brains of ASD patients.

*N*-ethylmaleimide-sensitive factor (NSF) is a homohexameric ATPase (Hanson et al., 1997; Fleming et al., 1998) that is an essential component of the protein machinery responsible for various membrane fusion events, including intercisternal Golgi protein transport and synaptic vesicle exocytosis (Rothman, 1994). NSF binds to soluble NSF attachment protein (SNAP)-receptor (SNARE) complexes and mediates the recycling of spent SNARE complexes for subsequent rounds of membrane fusion (Rothman, 1994; Hay and Scheller, 1997). While this is a major function of NSF, it also interacts with neurotransmitter receptors, such as AMPA receptors, and regulates their trafficking patterns or recycling (Nishimune et al., 1998; Osten et al., 1998; Song et al., 1998; Hanley et al., 2002; Evers et al., 2010). In addition to neurotransmitter receptors, we recently reported that NSF interacts with SERT under physiological conditions and is required for SERT membrane trafficking and its uptake function (Iwata et al., 2014). Notably, *NSF* mRNA expression is reduced in lymphocytes of ASD patients and is significantly correlated with the severity of clinical symptoms (Iwata et al., 2014).

Therefore, we hypothesized that NSF contributes to ASD pathophysiology through interactions with SERT and AMPA receptors and controls the trafficking and functions of these molecules. To test this hypothesis, we generated and evaluated Nsf heterozygous knockout (*Nsf* ^*+/-*^) mice by gene targeting. *Nsf* ^*+/-*^ mice exhibited a significant decrease in membrane SERT expression in the raphe and postsynaptic expression of AMPA receptors in the hippocampal CA1 region. We also found that *Nsf* ^*+/-*^ mice showed core ASD symptoms, such as abnormal sociability and communication, repetitiveness, and anxiety. In addition, *Nsf* ^*+/-*^ mice showed decreased postsynaptic density (PSD) areas and abnormal synaptic plasticity.

## Materials and Methods

### Animals

Four-week-old male mice were used for all experiments, except for the ultrasonic vocalization test (male pups at postnatal day 6). All experimental procedures were approved by the Animal Research Committee, University of Fukui, and the Institutional Animal Care and Use Committee of the Maebashi Institute of Technology. All experiments were conducted in compliance with institutional guidelines and regulations. All efforts were made to minimize the number of animals used and their suffering.

### Generation of *Nsf* Knockout Mice

**FIGURE 1:**
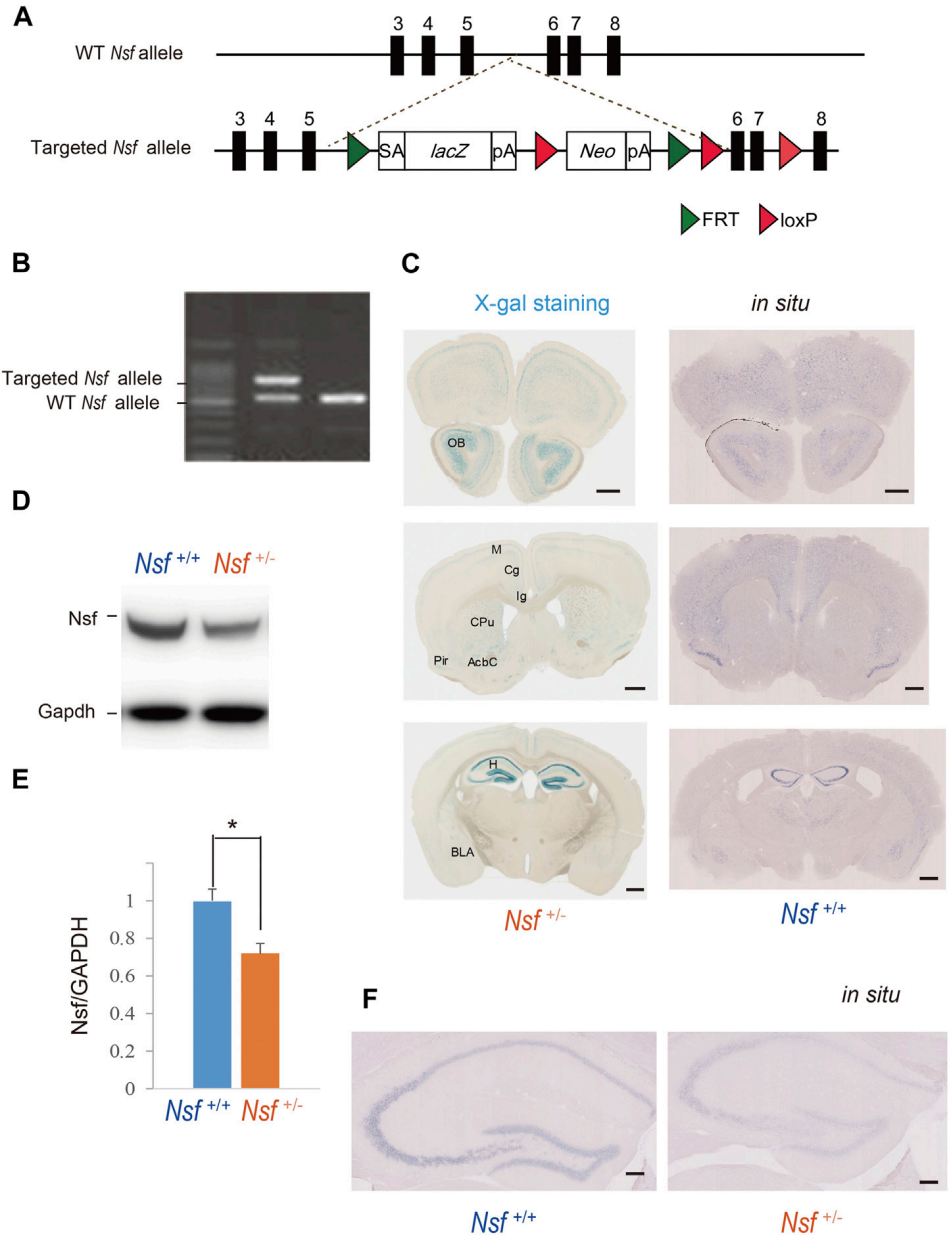
Generation of Nsf knockout mice **(A)** Schematic representation of the genomic structure of the relevant part of the *Nsf* wild-type (WT) allele and the targeted allele construct with splice acceptor (SA) sequence, *IRES* followed by *lacZ* (fusion of β-gal gene and neomycin phosphotransferase gene), and a polyadenylation signal sequence (pA). Exons 5 and 6 of *Nsf* are flanked by *lacZ* and neomycin resistance (*Neo*) cassettes. **(B)** WT alleles and targeted alleles were detected using genomic PCR. The WT and mutant alleles are shown as 504 and 737 bp fragments, respectively. **(C)** X-gal staining (left panels) and *in situ* hybridization of *Nsf* (right panels) in *Nsf* ^+/−^ mouse brains. H, hippocampus; M, motor cortex; Cg, cingulate cortex; Ig, indusium griseum; Cpu, caudate putamen; Pir, piriform cortex; Acb, accumbens nucleus; Bla, basolateral amygdala. Scale bar represents 500 μm. **(D)** Nsf protein expression in the hippocampus from *Nsf* ^+/+^ and *Nsf* ^+/−^ mice was detected by western blotting (upper panel). The blots were subsequently probed with an anti-Gapdh antibody as a protein loading control (lower panel). **(E)** Relative band densities of Nsf were quantified using scanning densitometry (*n* = 4 for each genotype; mean ± SEM. Student’s *t*-test, *p < 0.05). **(F)** *In situ* hybridization of Nsf in the hippocampus of *Nsf* ^*+/+*^ mice (right panel) and *Nsf^+/−^* mice (left panel). Scale bar represents 2.5 mm.

To generate *Nsf* knockout (KO) mice, we used C57BL/6N-background embryonic stem cells, EGR-101, carrying a “knockout first” (Testa et al., 2004) targeted *Nsf* allele obtained from the KOMP Repository (Vector ID: PG00174_Z_5_D06), which contains flippase recombination target-flanked *lacZ* and neomycin resistance (*Neo*) cassettes in front of a loxP-flanked (floxed) *Nsf* exon 6 (Figure 1A). The targeted *Nsf* allele was designed to be a knockout by splicing the cDNA into a *lacZ-neo* cassette. The cassette was then inserted upstream of a critical exon for *Nsf*, exon 6, to create a null allele of the gene. Embryonic stem cells were injected into eight-cell Institute for Cancer Research (ICR) mouse embryos, and chimeric blastocysts were transferred into the uteri of pseudo-pregnant ICR female mice (Fujihara et al., 2013). The resultant chimeric mice were bred to C57BL/6N background, and germline transmission was verified by conventional polymerase chain reaction (PCR) with the following primers for the wild-type allele, with a 504 bp fragment, (F: 5′-CCC​AGC​ATC​CTG​AAG​GGA-3′ in exon 6) and (R: 5′-CGA​TAA​GAT​TGA​GCG​ACG​AAT​TTT-3′ in exon 7), and the targeted allele, with a 737 bp fragment, (F: 5′-CCC​AGC​ATC​CTG​AAG​GGA-3′ in exon 6) and (R: 5′-ACT​GAT​GGC​GAG​CTC​AGA​CC-3′ in loxP), in F1 heterozygous KO mice (*Nsf* ^*+/-*^; Figure 1B). The mice were housed under specific pathogen-free conditions *and controlled laboratory conditions under an inverse 12* *h light/dark cycle* (lights on at 7:00 am)*, with ad libitum access to food and water.*

### X-gal Staining

Mice were transcardially perfused for 1 min with phosphate-buffered saline, followed by 12 min of perfusion with 0.1 M phosphate buffer containing 4% paraformaldehyde at a rate of 5 ml/min. After rapid removal of the brains from the skull, they were fixed in 4% paraformaldehyde overnight. Following a buffer change with 0.1 M phosphate buffer, the brain was sliced (100 µm thick) on a vibratome (Dosaka, Kyoto, Japan). After the brain tissue sections were immersed in NP40 deoxycholate solution (0.02% NP40, 0.01% deoxycholate) for 15 min, X-gal staining was performed by incubating the samples overnight at 37°C in X-gal solution (20 mg/ml X-gal/dimethylformamide [Sigma-Aldrich, MO, United States], 5 mM K_3_Fe(CN)_6_, 5 mM K_4_Fe(CN)_6_, 2 mM MgCl_2_, 0.02% NP-40, 0.01% sodium deoxycholate, 5 mM EDTA, and 1 × phosphate-buffered saline). The targeted vector included the bacterial beta-galactosidase reporter gene (*lacZ*) *sequence* and used the artificial substrate *X-gal*, which turns blue when cleaved by β-*galactosidase*. Nuclei were counterstained with Nuclear Fast Red (Sigma-Aldrich).

### Immunohistochemistry and Western Blotting

Experiments were performed according to a previously described method (Xie et al., 2019). Mouse anti-Nsf (123011 and 123002, Synaptic Systems, Göttingen, Germany), rabbit anti-HRP-Gapdh (M171-7, MBL, MA, United States), mouse anti-SERT (SC-1458, Santa Cruz Biotechnology, TX, United States), rabbit anti-GluA2 (MAB397, Millipore, MA, United States) and GluA1-3 antibodies were used (Xie et al., 2019).

### *In Situ* Hybridization

To confirm the expression pattern of *Nsf* in the mouse brain, we performed *in situ* hybridization using digoxigenin-labeled antisense RNA probes. The *Nsf* plasmid was prepared by pGEM-t kit PCR with the following primers: 5’-CGT​GAA​GTG​TCC​GCC​TCT-TAG​GCA​AAC​CAC​CCT​CCA-3’ and 5-CTT​GTC​TTT​AGC​TTC​AAT​GAT​AA-CGA​TAA​GAT​TGA​GCG​ACG​AA-3’. The subsequent experiments were performed according to previously described methods (Murata et al., 2020).

### Three-Chamber Test

The three-chamber testing apparatus consisted of a rectangular, three-chambered box and a lid with an infrared video camera (TimeCSI2; Ohara & Co., Tokyo, Japan). Each chamber was 20 cm × 40 cm × 22 cm, and the dividing walls were made of acrylic partitions, with small rectangular openings (5 × 3 cm) allowing access into each chamber. Small wire cages (9 cm radius × 22 cm height) were placed in both corners of the three-chambered box and illuminated at 50 lx. The wire cage consisted of vertical bars, allowing minimal contact among the mice to prevent fighting. The test was performed in six sessions (Tochitani et al., 2016). In session I, the subject mouse was placed in the middle chamber of an empty cage to habituate and freely investigate for 5 min. In session II, an unfamiliar C57BL/6N female mouse (stranger 1) was placed in one of the cages, and the subject mouse was allowed to explore the three chambers without restrictions for 5 min. In sessions III-V, the stranger 1 mouse was kept in the same cage, and unrestricted exploration by the subject mouse was allowed for 5 min. In session VI, a second unfamiliar C57BL/6N female mouse (stranger 2) was placed in the same cage, and the subject mouse was placed in the middle chamber and allowed to explore the chambers without restrictions for 5 min. Each mouse was used once per day. The movement of the subject mouse was recorded with an infrared video camera, and the time spent in close interaction in each wire cage was analyzed with ImageJ CSI software (Ohara & Co). The time spent in close interaction in each wire cage was converted into a preference index. The preference index in the sociability test was calculated as follows: ([time spent exploring the stranger mouse] − [time spent exploring the empty cage])/[total time spent exploring both targets] × 100 (Hisaoka et al., 2018).

### Ultrasonic Vocalization Task

Mouse pups (*n* = 26 in *Nsf* ^+/+^ mice and *n* = 20 in *Nsf* ^+/-^ mice) from different litters at postnatal day 6 were placed in an empty glass beaker in a sound attenuation recording chamber with an ultrasonic microphone (W500 × D350 × H350 mm). The frequency of the vocal sounds was observed using MKSPL software (Muromachi Kikai Co., Tokyo, Japan). Ultrasonic vocalizations from individual pups were recorded and analyzed for a period of 10 min using the Vocalization Analyzer software (Muromachi Kikai Co.). The same program was used to count all the calls above 30 kHZ.

### Open-Field Test

The open-field test was used to assess locomotor activity and repetitive behavior in a relatively large novel environment in a square arena (48 cm × 48 cm) (MELQUEST Co., Toyama, Japan; Tochitani et al., 2016). Mice were placed in the right-front corner of the open-field arena and allowed free movement for 30 min while being tracked by the SCANET MV-40 (Noldus Information Technology, Wageningen, Netherlands) automated tracking system. The total distance and vertical activity were automatically collected and analyzed using this system.

### Light/Dark Transition Test

The apparatus used for the light/dark transition test consisted of two boxes (15 cm × 15 cm × 15 cm), light and dark, each with a door (MELQUEST Co.). The light box illumination was 390 lx, whereas the dark box illumination was 2 lx. Mice were placed in the dark box, and the door was opened after initiating the test. The mice were allowed to move freely between the two chambers with the door open for 10 min.

### Slice Biotinylation

Slice biotinylation was performed as previously described (Gill et al., 2011). Mouse hippocampal and midbrain slices (400 µm in thickness) were incubated in slicing buffer (124 mM NaCl, 26 mM NaHCO_3_, 3 mM KCl, 10 mM glucose, 0.5 mM CaCl_2_, and 4 mM MgCl_2_) for 30 min and then recovered in biotinylation solution (124 mM NaCl, 26 mM NaHCO_3_, 3 mM KCl, 10 mM glucose, 2.3 mM CaCl_2_, and 1.3 mM MgCl_2_) for 30 min at 20–25°C. Slices were then preincubated in ice-cold biotinylation solution for 1 min. Surface proteins of the dissected tissue were labeled with sulfo-NHS-SS-biotin (1.5 mg/ml; Pierce) for 30 min on ice, and the reaction was quenched with biotinylation solution with 50 mM glycine three times. Slices were homogenized with Tris buffer (50 mM Tris, pH 7.4, 2 mM EGTA) and then sonicated. To isolate the membrane fraction, homogenates were centrifuged at 100,000 × *g* for 20 min, and the pellet was resuspended in RIPA buffer (50 mM Tris, pH 7.4, 1 mM EDTA, 2 mM EGTA, 150 mM NaCl, 1% NP40, and 0.5% DOC) for 30 min. The lysate was cleared by centrifugation at 100,000 × g for 20 min. High-capacity streptavidin agarose resins (Roche, Basel, Switzerland) were added and incubated at 4°C for 2 h. Non-bound internal protein solution was removed. Beads were washed with RIPA buffer and biotinylated surface proteins were eluted by boiling for 10 min in Laemmli buffer containing dithiothreitol (7.7 mg/ml). Eluted and internal proteins were detected using western blotting. Western blots were carried out using 10% Tris-glycine extended Stain-Free gradient gels (Bio-Rad, CA, United States) and subsequently transferred to nitrocellulose membranes (Bio-Rad). Gels were activated by UV exposure for 2 min using a ChemiDoc™ MP imager (Bio-Rad). The membranes were imaged for Stain-Free staining, and total protein was quantified using ImageLab 5.2.1 (Bio-Rad).

### SDS-Digested Freeze-Fracture Replica Immunolabeling

“Brain slices (130 μm) were prepared from the hippocampi of post-natal day 28 mice for FRIL. Mice were perfused transcardially for 1 min with PBS, followed by 12 min of perfusion with 0.1 M PB containing 2% paraformaldehyde and 15% saturated picric acid solution at a rate of 5 ml/min. The brains were quickly removed from the skull and sliced (130 µm thick) on a vibratome (Dosaka, Kyoto, Japan). Hippocampal slices were cryoprotected in 30% glycerol in 0.1 M PB and high-pressure frozen using HPM010 machine (Bal-Tec, Balzers, Liechtenstein). The frozen slices were then freeze fractured at −130°C and replicated with an initial carbon layer (5 nm), shadowed unidirectionally with platinum (2 nm), and strengthened with a second carbon layer (15 nm) in a BAF060 freeze-etching machine (Bal-Tec). After thawing, the tissue attached to the replicas was solubilized by shaking at 80°C for 18 h in the following solubilisation solution: 15 mM Tris [hydroxymethyl]-aminomethane, 20% sucrose, and 2.5% sodium dodecyl sulfate, pH 8.3. Immunolabelling of replicas was carried out according to previously published procedures with minor modifications46. Blocking was performed with a solution consisting of 5% bovine serum albumin and 0.1% TWEEN 20 in TBS (pH 7.4). The replicas were incubated in primary antibodies (anti-GluA1-3 or anti-GluA1 antibodies, both generated in horse against synthetic peptides deduced from the common and unique aa sequences of the extracellular portion of GluA1, respectively) at 15°C for 3 days. The specificity of these antibodies in FRIL analysis was confirmed by the absence of labelling in parallel fibre-Purkinje cell synapses of GluA2/3 knock-out mice and hippocampal synapses of GluA1 knock-out mice. Following extensive washing with unbound primary antibody, the replicas were incubated with gold-conjugated anti-rabbit secondary antibodies (British Biocell International, Cardiff, United Kingdom; 5 nm), overnight at 15°C. To mark IMP clusters on exoplasmic-face derived from excitatory synapses, NMDA receptor labelling was carried out simultaneously with the secondary antibody incubation by adding mouse anti-NR1 antibody (clone 54.1, 1:100, Millipore), which was then detected by incubation with anti-mouse secondary antibodies (British Biocell International; 10 nm) at room temperature for 1 h. The replicas were then mounted on pioloform-coated copper mesh grids and examined at 80 kV acceleration voltage in an H-7650 transmission electron microscope equipped with a CCD camera (Hitachi High-Technologies Corporation, Tokyo, Japan). Electron micrographs captured at 400,00x were analysed with the program ImageJ (Rasband, W.S., ImageJ, U.S. National Institutes of Health, Bethesda, MA, http://imagej.nih.gov/ij/, 1997–2015) for measurement of synaptic area and quantification of immunogold particles within individual synapses. (Xie et al., 2019).”

### Ultrastructural Reconstructions

Experiments were performed according to our previously described methods (Xie et al., 2019). PSD area was identified as the membrane facing PSD which is clearly identified as an electron-dense thickening in dendritic spines. Independent traces were drawn for the entire spine structure and PSD and three-dimensional area were obtained. Three-dimensional reconstruction of dendritic spines was carried out with the aid of reconstruct software (Reconstruct 1.1.0.0, available from https://synapseweb.clm.utexas.edu).

### Electrophysiology

A glass microelectrode (Narishige, Tokyo, Japan) filled with artificial cerebrospinal fluid (ACSF, 2–4 MΏ electrical resistance) was used. Field excitatory postsynaptic potentials (fEPSPs) were recorded in the CA1 stratum radiatum with the glass microelectrode. For experiment of input-output relationship, the input-output curve of fEPSP slope (mV/ms) versus presynaptic fiber volleys (FV; mV) at the Schaffer collateral pathway was observed in slices. Paired-pulse facilitation, the short-term enhancement of synaptic efficacy following delivery of two closely spaced stimuli (inter-pulse interval; 25–500 ms), was also assessed. We induced long-term potentiation (LTP) with 100 pulses applied at a rate of 100 Hz for 1 s and long-term depression (LTD) with 900 pulses applied at a rate of 1 Hz for 15 min (Ishikawa et al., 2011). Hippocampal slice preparation and electrophysiology were performed according to our previously described methods (Xie et al., 2019).

### Statistical Analysis

For All statistical analyses were performed using IBM SPSS Statistics 23 and JMP Pro 14. Pairwise comparisons between groups were conducted using the two-tailed Student’s *t-*test or Mann–Whitney *U test*, and correlations were tested for statistical significance using Pearson’s correlation test or Spearman’s rank-order test. A two-way repeated-measures ANOVA with Tukey’s *post hoc* test was used for the analysis of data from the social interaction test. The null hypothesis was rejected at *p* < 0.05.

## Results

### Generation of *Nsf* KO Mice

To elucidate NSF involvement in the onset and/or pathophysiology of ASD, we generated *Nsf* KO mice using the International Knockout Mouse Consortium targeting vector inserted between exons 5 and 6 of *Nsf* (Figure 1A). The targeted *Nsf* allele was designed to be a KO by splicing the cDNA into the *lacZ*-*neo* cassette, which was inserted upstream of a critical exon for *Nsf*, i.e., exon 6 (Figure 1A). The targeted allele of the founder (*Nsf* ^+/-^ mice) was confirmed by PCR (Figure 1B). Because of the *lacZ* cassette in the targeted allele, cells with the targeted allele were detected by X-gal staining (West et al., 2015). We found strong lacZ expression in the olfactory bulb and hippocampus and moderate expression in the cortex, striatum, and amygdala in *Nsf* ^+/-^ mice (Figure 1C, left panels). In contrast, no signals were detected in *Nsf* ^+/+^ mice (Supplementary Figure S1A). The X-gal staining patterns were identical to endogenous *Nsf* expression patterns confirmed by *in situ* hybridization (Figure 1C, right panels), indicating that the *Nsf* ^+/-^ mouse model was successfully established. Since homozygous KO mice (*Nsf* ^*−/−*^) caused early embryonic lethality, we used heterozygous KO mice (*Nsf* ^*+/-*^) in all experiments in this study. *Nsf* ^*+/-*^ mice were born in good health and grew into adulthood. There were no notable differences in body and brain weights between *Nsf* ^*+/+*^ and *Nsf* ^*+/-*^ mice (Supplementary Figure S2). In *Nsf* ^*+/-*^mouse brains, Nsf expression decreased by an average of 72% compared with that of *Nsf* ^*+/+*^ mouse brains (*Nsf* ^*+/+*^ mice, 1.00 ± 0.07; *Nsf* ^*+/-*^ mice, 0.72 ± 0.05, *p = 0.03, Student’s *t*-test) (Figures 1D,E). We examined the expression pattern of Nsf in *Nsf* ^*+/-*^mice using immunofluorescence. In support of the western blotting results, Nsf expression was decreased without changing the expression pattern itself (Figure 1F and Supplementary Figure S1B).

### *Nsf* ^+/-^ Mice Showed Abnormalities in Social Interaction and Communication

**FIGURE 2:**
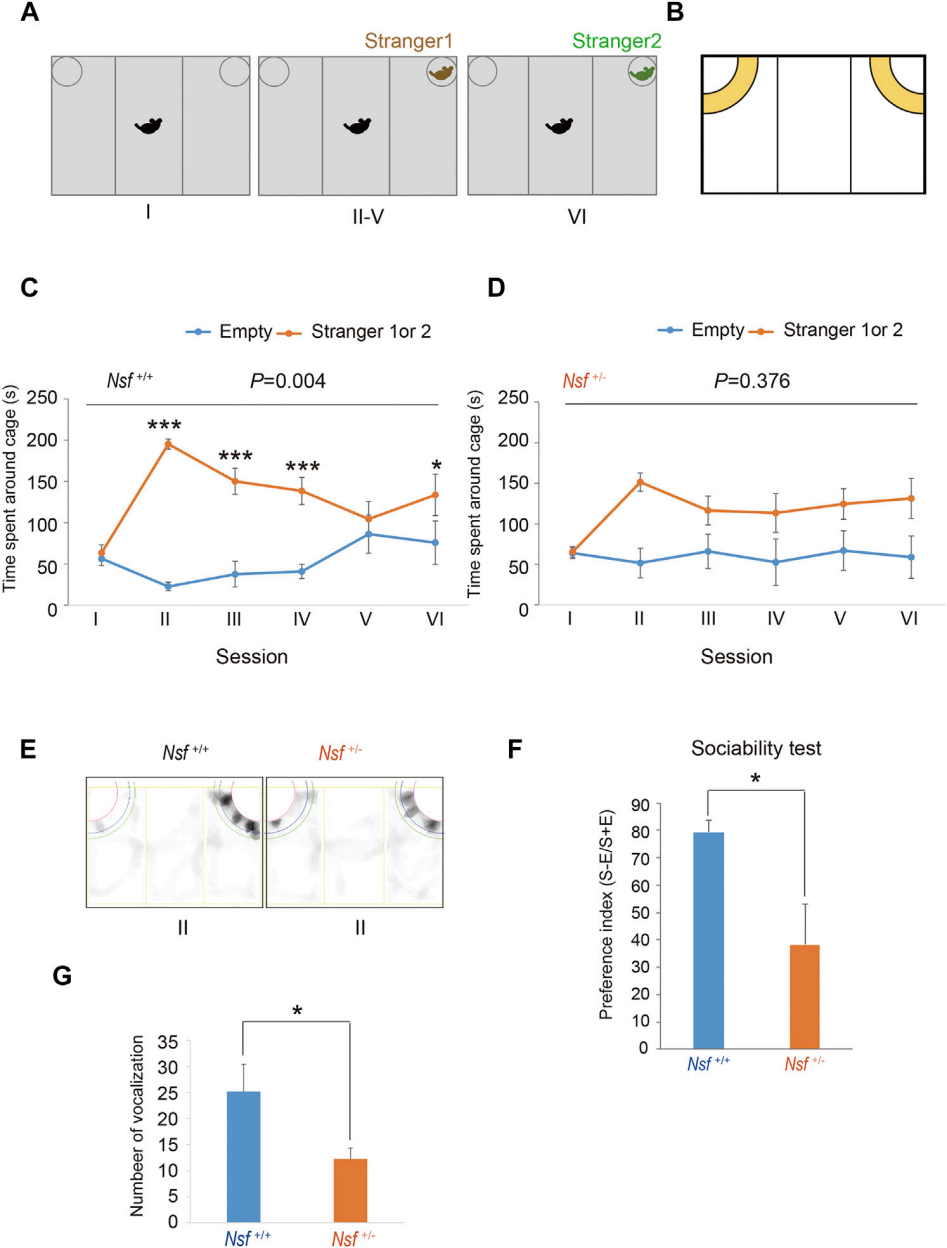
Deficits in social interaction and communication in *Nsf^+/−^* mice. **(A)** Schematic representation of three-chamber social interaction tests. Session I: the subject mouse was placed in the middle chamber without any stimulants. Session II–V: stranger 1 was placed inside a cage located in one of the chambers. Then, the subject mouse was placed at the center of the middle chamber. The subject mouse investigated the same stimulant (stranger) mouse during each of the four sessions (II–V). Session VI: stranger 2, instead of stranger 1, was placed inside the same cage as session II-V. Then, the subject mouse was placed at the center of the middle chamber. The subject mouse moved freely in the three chambers for 5 min for each session. **(B)** Yellow represents the virtual interaction areas near cages. **(C)** Time spent around cages with “stranger 1 or 2” or “Empty” by *Nsf^+/+^* mice during sessions I–IV (*n* = 13, mean ± SEM. Two-way repeated measures ANOVA, *p* = 0.004, Tukey’s post hoc test, ***p < 0.001, *p < 0.05). **(D)** Time spent around cages with “stranger 1 or 2” or “Empty” by *Nsf^+/−^* mice during sessions I–IV (*n* = 13, Mean ± SEM. Two-way repeated measures ANOVA, *p* = 0.376). **(E)** Representative mount-graph of each genotype during session II. **(F)** The preference index of the sociability (*n* = 13 for each genotype, mean ± SEM. Wilcoxon/Kruskal-Wallis test, *p < 0.05). **(G)** Number of ultrasonic vocalizations emitted by postnatal day 6 pups of each genotype during a 10 min separation from their mother (*n* = 26 in *Nsf^+/+^* mice and *n* = 20 in *Nsf^+/−^* mice, mean ± SEM. Spearman’s rank-order test, *p < 0.05).

We evaluated the effect of *Nsf* downregulation on mouse behavior. First, social interaction was assessed by a three-chamber test with six sessions (Tochitani et al., 2016). Following habituation (session I), mice were introduced into the center of the box, which contained a cage with a stranger (stimulus) mouse in a corner (session II), and the session was repeated three times with the same stimulus mouse (session III-V; Figure 2A). In session VI, we introduced the stranger mouse to a new stimulus mouse (Figure 2A). *Nsf* ^*+/+*^ mice spent significantly more time around the cage containing a stranger mouse than around the empty cage in sessions II, III, and IV (*p* < 0.01, two-way repeated measures ANOVA; session I, *p* = 0.776; sessions II, III, and IV, *p* < 0.001; session V, *p* = 0.453, Tukey’s post hoc test; Figures 2B,C). In session V, *Nsf* ^*+/+*^ mice showed a decline in the time spent around the cage containing a stranger mouse because stimuli became familiar, and the presentation of an unfamiliar mouse in session VI resulted in significantly more time spent around the cage containing a stranger mouse than around the empty cage (session VI, *p* = 0.020, Tukey’s post hoc test; Figure 2C). In contrast, *Nsf* ^*+/-*^ mice did not show a preference for social targets throughout testing (*p* = 0.376, two-way repeated measures ANOVA; Figure 2D). In addition, *Nsf* ^*+/-*^ mice showed significantly less interaction with the stranger than *Nsf* ^*+/+*^ mice during session II (*Nsf* ^*+/-*^ mice, 38.3 ± 14.7; *Nsf* ^*+/+*^ mice, 79.4 ± 4.5, Wilcoxon/Kruskal-Wallis test, *p = 0.02; Figures 2E,F). Separation-induced ultrasonic vocalizations were measured to evaluate the communication abilities of *Nsf* ^*+/-*^ mice. Ultrasonic calls are important for mother–infant social interactions (Smotherman et al., 1974) and represent important neurobehavioral development markers (Branchi et al., 1998). At postnatal day 6, *Nsf* ^+/-^ pups emitted significantly fewer calls than *Nsf* ^+/+^ pups (*Nsf* ^+/+^ mice, 25.2 ± 12.3; *Nsf* ^+/-^ mice, 12.3 ± 2.1, *p = 0.04. Spearman’s rank-order test; Figure 2G).

### *Nsf* ^+/-^ Mice Showed Increased Repetitive Behavior and Anxiety

**FIGURE 3:**
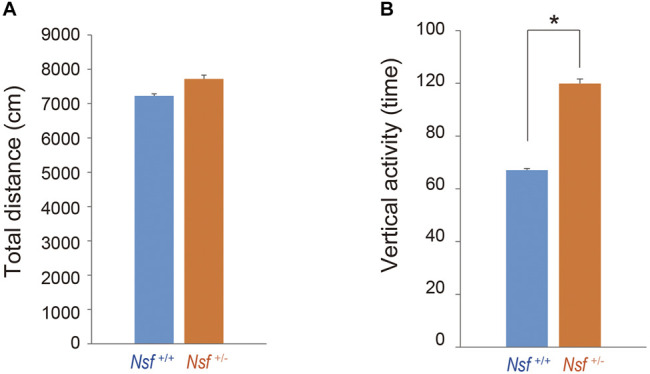
Increase in repetitive behavior of *Nsf^+/−^* mice in the open field. **(A)** Total distance (locomotor activity) and **(B)** vertical activity (repetitive behavior) of *Nsf^+/+^* and *Nsf^+/−^* mice. (*Nsf^+/−^* mice, *n* = 13, *Nsf^+/+^* mice, *n* = 11, mean ± SEM. Student’s t-test.*p < 0.05).

**FIGURE 4:**
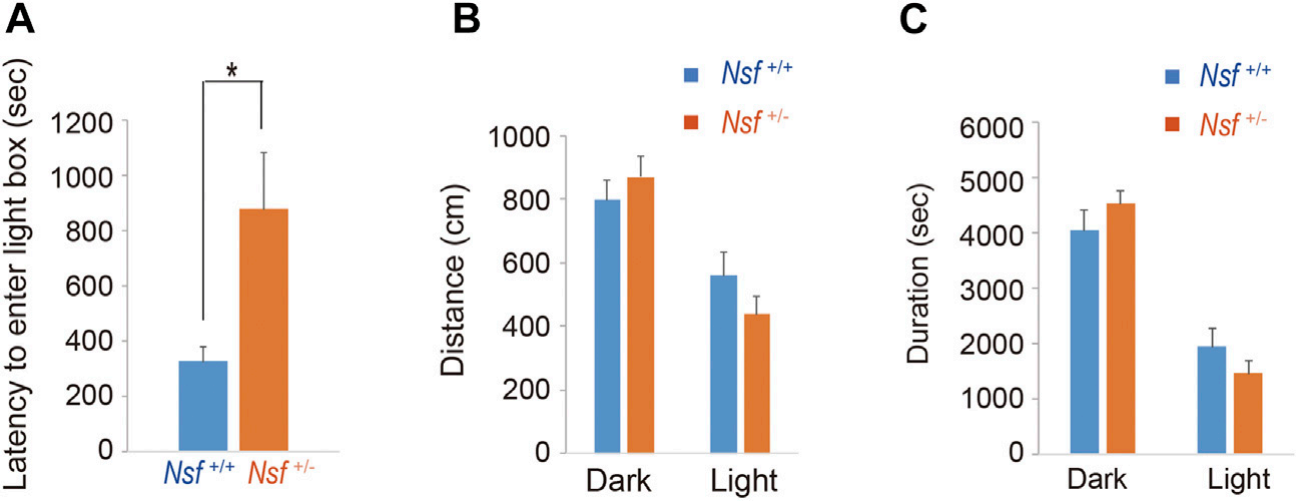
*Nsf^+/−^* mice show anxiety in the light/dark box test. **(A)** The latency to the first entry of the light box. **(B)** The total distance between the light and dark boxes. **(C)** The time spent in the light and dark boxes. (*n* = 11 for each genotype, mean ± SEM. Student’s t-test. *p < 0.05).

We measured the locomotor activity and relative behavior of *Nsf* ^+/-^ mice in a novel environment using an open-field test. There was no significant between-group difference in the total distance (*Nsf* ^+/+^ mice, 7,226.3 ± 444.6; *Nsf* ^+/-^ mice 7,719.6 ± 310.5, *p* = 0.37, Student’s *t*-test; Figure 3A). In contrast, vertical activity (that is, repetitive behavior) were significantly increased in *Nsf* ^+/-^ mice compared with *Nsf* ^+/+^ mice (*Nsf* ^+/+^ mice, 67.1 ± 10.7; *Nsf* ^+/-^ mice 99.9 ± 8.8, *p = 0.04, Student’s *t*-test; Figure 3B). Next, to assess anxiety behavior, we conducted a light/dark box test. The latency to enter the light chamber served as an anxiety index, and *Nsf* ^+/-^ mice exhibited a longer latency to enter the light box than *Nsf* ^+/+^ mice (*Nsf* ^+/+^ mice, 324.0 ± 54.3; *Nsf* ^+/-^ mice, 878.8 ± 205.8; Student’s *t*-test, *p = 0.02; Figure 4A). The distance (dark box: *Nsf* ^+/+^ mice, 796.9 ± 59.8; *Nsf* ^+/-^ mice, 869.3 ± 64.7, *p* = 0.44. light box: *Nsf* ^+/+^ mice, 560.9 ± 71.59; *Nsf* ^+/-^ mice, 438.4 ± 56.3, *p* = 0.21, Student’s *t*-test; Figure 4B) and duration (dark box: *Nsf* ^+/+^ mice, 4,052.5 ± 355.3; *Nsf* ^+/-^ mice, 4,538.3 ± 244.7, *p* = 0.30. light box: *Nsf* ^+/+^ mice, 1,932.1 ± 355.0; *Nsf* ^+/-^ mice, 1,449.8 ± 246.0, *p* = 0.30, Student’s *t*-test; Figure 4C) in the dark and light boxes were not significantly different for either mouse.

### Decrease in Serotonin Transporter Surface Expression in the Raphe of *Nsf* ^+/-^ Mice

**FIGURE 5:**
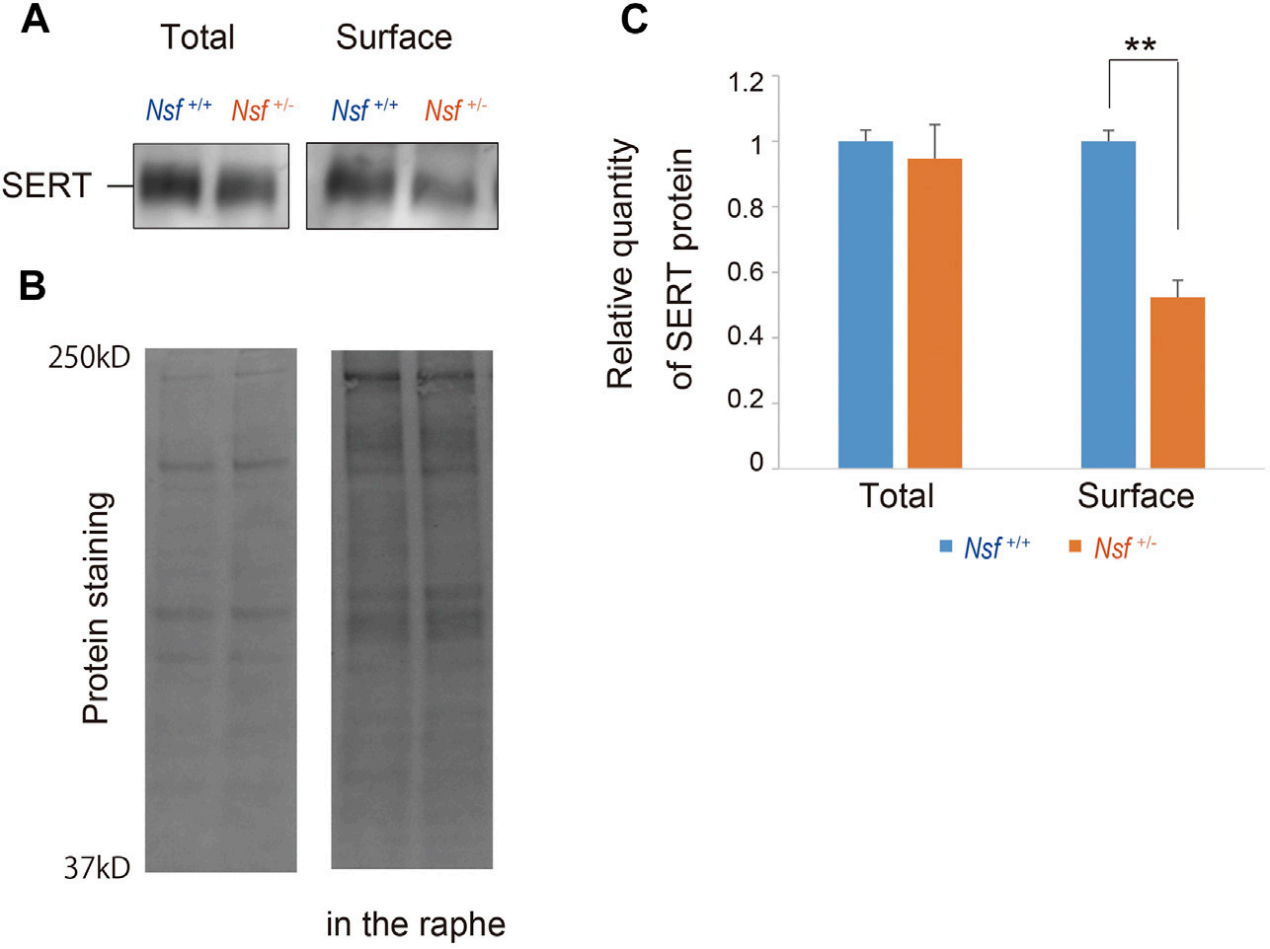
Decrease in serotonin transporter (SERT) expression at the membrane surface in *Nsf^+/−^* mice. **(A)** Total and biotinylated membrane protein levels in the raphe of mouse of each genotype were analyzed by immunoblotting using an anti-SERT antibody. **(B)** Stain-Free gels were activated by UV exposure and imaged using a ChemiDocTM MP imager as protein loading control. **(C)** Relative band densities of SERT were quantified using scanning densitometry and normalized to protein loading control Results are expressed as a ratio of *Nsf^+/+^* mice expression, resulting in a *Nsf^+/+^* mice ratio of 1. (*n* = 4 for each genotype, mean ± SEM. Student’s t-test, **p < 0.01).

We previously demonstrated that Nsf bound to SERT *in vitro* and vivo (Iwata et al., 2014). In addition, Nsf co-localized with SERT in the raphe of *Nsf^+/+^* mice (Supplementary Figure S3). Our previous study also demonstrated that Nsf was important for SERT membrane trafficking *in vitro* (Iwata et al., 2014). To confirm this *in vivo*, we compared SERT surface levels in the raphe from *Nsf* ^+/+^ and *Nsf* ^+/-^ mice using a cell-impermeant biotinylation reagent. In support of our *in vitro* data, SERT surface expression was decreased by an average of 48% in the raphe of *Nsf* ^+/-^ mice compared with that of *Nsf* ^+/+^ mice, despite the total SERT expression remaining unchanged (SERT surface expression: *Nsf* ^+/+^ mice, 0.31 ± 0.02; *Nsf* ^+/-^ mice, 0.15 ± 0.02, **p = 0.004. Total SERT expression: *Nsf* ^+/+^ mice, 1.29 ± 0.04; *Nsf* ^+/-^ mice, 1.22 ± 0.13, *p* = 0.690, Student’s *t*-test; Figures 5A,C). As a protein loading control, Stain-Free gels were activated by UV exposure and imaged using a ChemiDoc™ MP imager (Figure 5B).

### Nsf Contributes to GluA1-3 Accumulation at the Synaptic Membrane Surface

**FIGURE 6:**
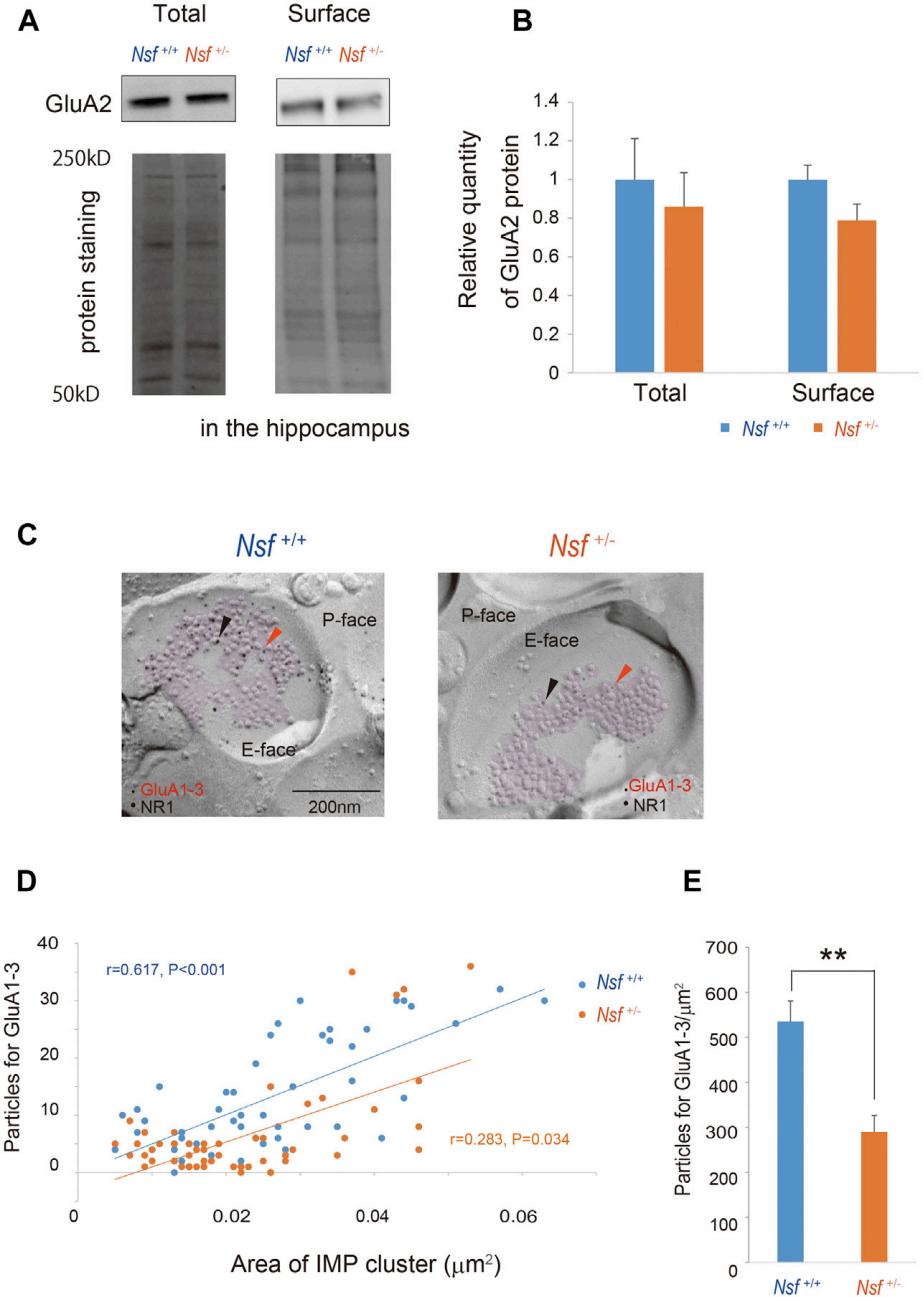
Decrease in alpha-amino-3-hydroxy-5-methyl-4-isoxazole propionic acid (AMPA) receptor expression in postsynaptic membrane in *Nsf^+/−^* mice. **(A)** Total and biotinylated membrane protein levels in the hippocampus of mice of each genotype were analyzed by immunoblotting using anti-AMPA receptor (GluA) 2 antibody. Stain-Free gels were activated by UV exposure and imaged using a ChemiDocTM MP imager as a protein loading control. **(B)** Relative band densities of GluA2 were quantified using scanning densitometry. and normalized to protein loading control Results are expressed as a ratio of *Nsf^+/+^* mice expression, resulting in a *Nsf^+/+^* mice ratio of 1. (*n* = 4 for each genotype, mean ± SEM. Student’s t-test). **(C)** Replicas were prepared from the hippocampal CA1 region. Using a transmission electron microscope, postsynaptic membrane specializations of excitatory synapses in replicas were identified in the exoplasmic (E)-face of the plasma membrane by clusters of intra-membrane particles (IMP clusters, purple). Immunoreactivity for GluA1-3 was visualized with 5 nm immunogold particles (orange arrowheads). Immunolabeling for the NR1 subunit was visualized with 10 nm immunogold particles (black arrowheads) to confirm the IMP cluster areas. **(D)** The numbers of immunoparticles for GluA1-3 in individual IMP clusters were plotted against the IMP cluster areas. Correlation between the GluA1-3 labeling number and synaptic area in mice of each genotype (Spearman’s rank-order test: the *Nsf^+/+^* mice, *n* = 56 synapses, *r* = 0.617, *p* < 0.001; the *Nsf^+/−^* mice, *n* = 56 synapses, *r* = 0.283, *p = 0.034). **(E)** The average labeling particles for synaptic GluA1-3 in *Nsf^+/−^* and *Nsf^+/+^* mice (mean ± SEM. Spearman’s rank-order test, **p < 0.01).

We focused on the hippocampus in the central nervous system because Nsf expression is the highest in this brain region (Puschel et al., 1994; Figure 1C). Indeed, *in vitro* studies have shown that Nsf interacts with GluA2 and regulates the surface expression of GluA2-containing AMPA receptors in hippocampal neurons (Nishimune et al., 1998; Noel et al., 1999; Lu et al., 2014). Here, we examined whether *Nsf* haploinsufficiency changed GluA2 membrane expression *in vivo*. Using a cell-impermeant biotinylation reagent, we compared GluA2 surface levels in the hippocampus of *Nsf* ^+/+^ and *Nsf* ^+/-^ mice. Total GluA2 was not changed, and unexpectedly, GluA2 surface expression was not changed significantly in *Nsf* ^+/-^ mice (total expression: *Nsf* ^+/+^ mice, 0.07 ± 0.01; *Nsf* ^+/-^ mice, 0.06 ± 0.01, *p* = 0.673. GluA2 surface expression: *Nsf* ^+/+^ mice, 0.14 ± 0.02; *Nsf* ^+/-^ mice, 0.11 ± 0.02, *p* = 0.336. Student’s *t*-test; Figures 6A,B). Nsf has been previously reported to be highly expressed in the PSD (Walsh and Kuruc, 1992). Therefore, we examined whether a decrease in Nsf levels affected AMPA receptor expression, including GluA2, at the postsynaptic membrane. Using the FRIL technique (Xie et al., 2019), we monitored endogenous AMPA receptor (GluA1-3) expression at the surfaces of the stratum radiatum spines of the hippocampal CA1 region. We could not measure GluA2 levels specifically, as there was no appropriate anti-GluA2 antibody available for this technique. The subcellular localization of the postsynaptic membrane area in a dendritic spine was identified in the exoplasmic face of the replicas as an area accompanied by clustered intramembrane particles (IMP) labeled for the NR1 subunit of the NMDA receptor (Figure 6C). The number of immunogold particles for GluA1-3 in individual IMP cluster areas was proportional to the area of the IMP clusters in both *Nsf* ^+/+^ and *Nsf* ^+/-^ mice (*Nsf* ^+/+^ mice, *r* = 0.617, *p* < 0.001; r = 0.283, *Nsf* ^+/-^ mice, *p* = 0.034, Spearman’s rank-order test; Figure 6D). In contrast, a significant reduction of 54% in the labeling density for synaptic GluA1-3 was observed in *Nsf* ^+/-^ mice compared with *Nsf* ^+/+^ mice (*Nsf* ^+/+^ mice, 535.5 ± 45.5, gold particles/µm^2^; *Nsf* ^+/-^ mice, 289.7 ± 36.8, gold particles/µm^2^, Spearman’s rank-order test, **p = 0.005; Figure 6E).

### Postsynaptic Density Areas Are Decreased in *Nsf* ^+/-^ Mice

**FIGURE 7:**
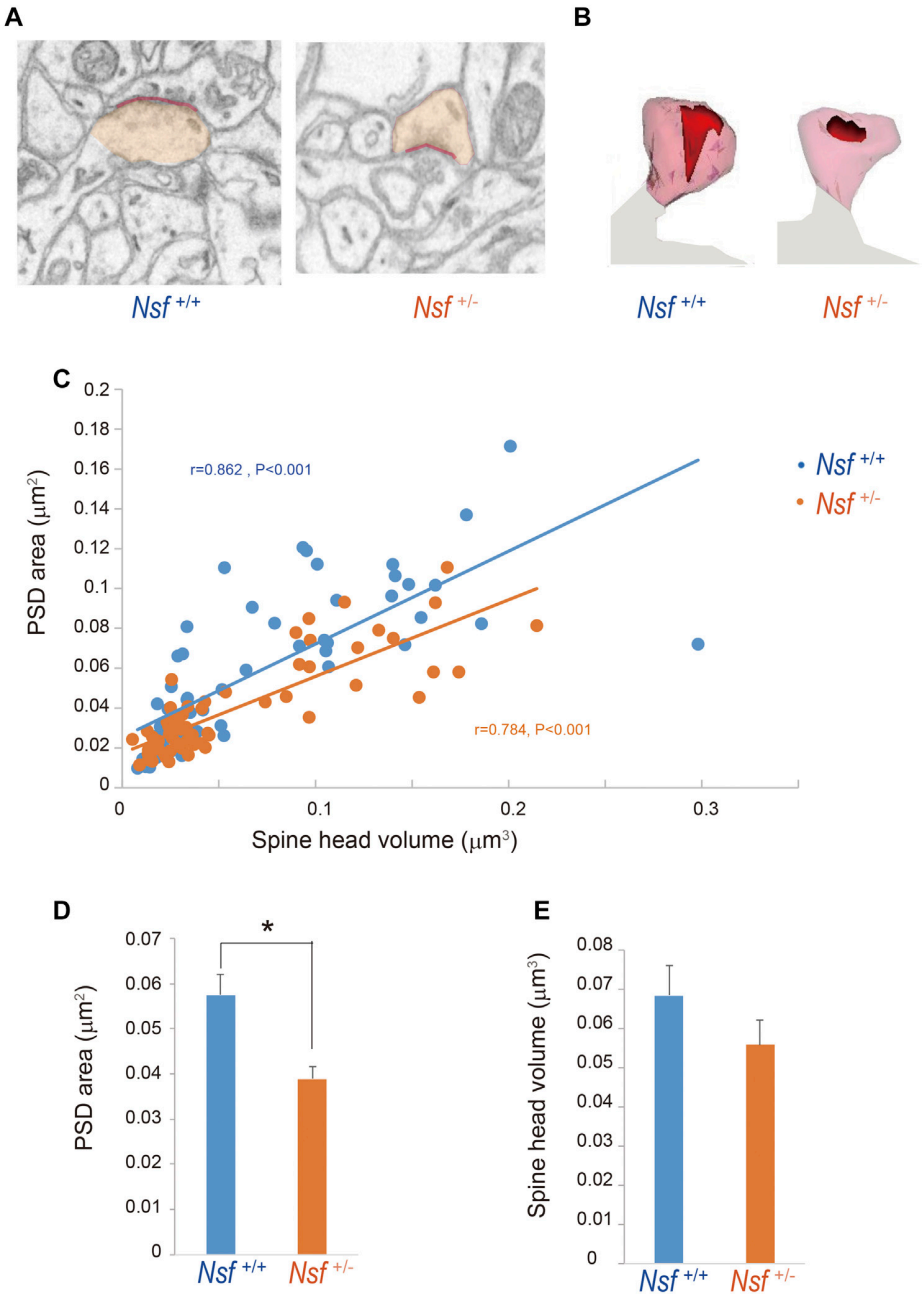
Decrease in postsynaptic density (PSD) area in postsynaptic membrane in *Nsf^+/−^* mice. Reconstruction of dendritic spines from serial FIB-SEM images. PSD was defined as postsynaptic membrane specialization. **(A)** Representative FIB-SEM images of the hippocampal CA1 region of *Nsf^+/+^* and *Nsf^+/−^* mice (spine head in orange and PSD in red). **(B)** Examples of 3D-reconstructed spines from *Nsf^+/+^* and *Nsf^+/−^* mice (head in transparent orange and PSD in red). **(C)** Correlation between the PSD area and spine head volume in mice of each genotype (Spearman’s rank-order test: *Nsf^+/+^* mice, *n* = 58 synapses, *r* = 0.862, *p* < 0.001; *Nsf^+/−^* mice, *n* = 65 synapses, *r* = 0.784, *p* < 0.001). **(D)** The average PSD areas and **(E)** head volumes in the hippocampus of *Nsf^+/+^* and *Nsf^+/−^* mice (mean ± SEM. Spearman’s rank-order test, *p* = 0.074).

To investigate whether the decrease in GluA1-3 levels at the synaptic membrane surface of *Nsf* ^+/-^ mice was due to an enlargement of PSD areas in the mutant mice, we reconstructed spines from serial electron micrographs captured using a focused ion beam scanning electron microscope (FIB-SEM). PSDs were observed as electron-dense thickenings of the postsynaptic plasma membrane, which was similar to their appearance in conventional transmission electron microscopy (Figure 7A). The PSD region was traced with a red line in individual images, and the entire area of the postsynaptic membrane specialization as well as the spine head (orange) was reconstructed (Figures 7A,B). The area of the PSD was proportional to the volume of the spine head in both genotypes (*r* = 0.862, *p* < 0.001 for *Nsf* ^+/+^ mice and *r* = 0.784, *p* < 0.001 for *Nsf* ^+/−^ mice; Figure 7C). The average PSD areas were decreased in *Nsf* ^+/-^ mice (0.057 ± 0.004 μm^2^ for *Nsf* ^+/+^ mice and 0.039 ± 0.001 μm^2^ for *Nsf* ^+/-^ mice, Spearman’s rank-order test, *p = 0.049; Figure 7D), whereas there was no significant difference between the two genotypes in spine head volumes (0.068 ± 0.008 μm^3^ for *Nsf* ^+/+^ mice and 0.056 ± 0.006 μm^3^ for *Nsf* ^+/-^ mice, Spearman’s rank-order test, *p* = 0.074; Figure 7E).

### Nsf Is Required for Normal Induction of Synaptic Plasticity Long-Term Depression but Not Long-Term Potentiation Induction

**FIGURE 8:**
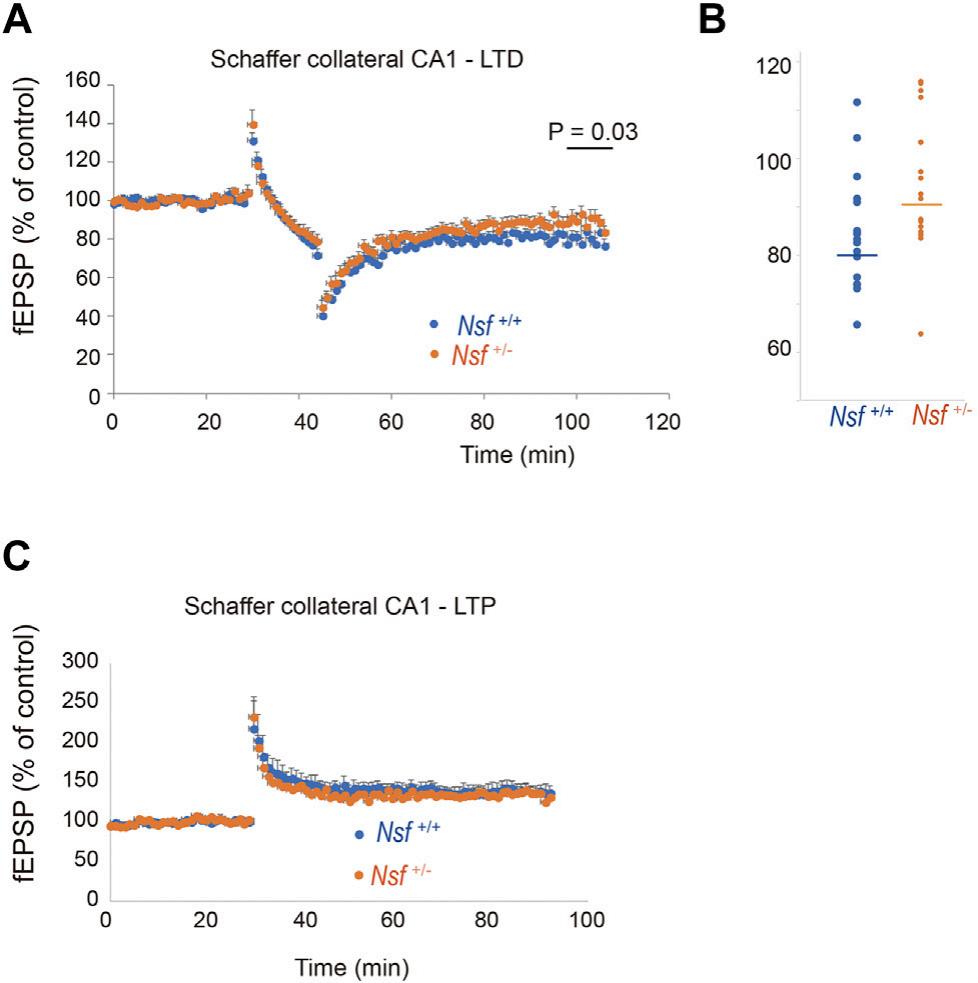
Nsf is required for normal induction of long-term depression (LTD). **(A)** Electrophysiological analyses of the effects of Nsf on hippocampal LTD. LTD was induced by low-frequency stimulation (LFS; 1 Hz; 900 pulses, 15 min) of the Schaffer collaterals in hippocampus slices from *Nsf^+/−^* and *Nsf^+/+^* mice (*n* = 17 slices for each mouse. Mean ± SEM. Mann–Whitney U test, *p* = 0.031 at the boxed time points, 97–106 min). A value of 100% corresponds to the pre-LFS baseline. **(B)** The dispersion of individual fEPSP data for *Nsf^+/+^* and *Nsf^+/−^* mice at the significant time point (97–106 min) in **(A)**. **(C)** Electrophysiological analyses of the effects of Nsf on hippocampal long-term potentiation (LTP). LTP was induced by high-frequency stimulation (HFS) (100 Hz; 100 pulses, 1 s) of the Schaffer collaterals from *Nsf^+/−^* and *Nsf^+/+^* mice (*n* = 6 slices for *Nsf^+/−^* mice and *n* = 9 slices for *Nsf^+/−^* mice. Mean ± SEM. Student’s t-test, *p* = 0.77). A value of 100% corresponds to the pre-LFS baseline.

Synaptic AMPA receptors have been suggested to be important for synaptic plasticity, such as LTP and LTD (Luscher et al., 2000; Carroll et al., 2001; Sheng and Lee, 2001; Malenka, 2003). Therefore, we examined whether LTP and LTD induction were influenced by decreased levels of AMPA receptors in *Nsf* ^+/-^ mice. LTD was induced in hippocampal CA1 neurons by low-frequency stimulation of Schaffer collaterals. Expectedly, elevation of recorded fEPSPs was decreased in *Nsf^+/−^* mice compared to *Nsf^+/+^* mice at the indicated time points (97–106 min). (Mann–Whitney U test, *p = 0.031; Figures 8A,B). We also induced LTP by high-frequency stimulation of Schaffer collaterals in both mice and observed no change in the fEPSPs (Figure 8C). In addition, we analysed input-output relationship and paired-pulse facilitation (PPF) to assess the strength of basal synaptic transmission and the presynaptically mediated form of potentiation, respectively. No significant difference was observed in terms of basal synaptic function between the *Nsf^+/+^* mice and the *Nsf^+/−^* mice (Supplementary Figures S4A,B).

## Discussion

A decrease in NSF expression has been suggested in individuals with ASD (Iwata et al., 2014); however, causality between NSF expression and the onset and/or pathophysiology of ASD remains unclear. In this study, we first generated *Nsf* ^+/-^ mice and found that the mice showed core ASD symptoms, such as abnormal sociability and communication, repetitiveness, and anxiety. Additionally, these mice showed decreased membrane expression of SERT and AMPA receptors in the brain, which were hound in ASD patients (Purcell et al., 2001a; Nakamura et al., 2010; Iwata et al., 2014). The mice also showed impaired PSD and LTD in the hippocampal CA1 region.

### Decrease in Membrane Expression of Serotonin Transporter and Alpha-Amino-3-Hydroxy-5-Methyl-4-Isoxazole Propionic Acid Receptors in *Nsf* ^+/-^ Mice

Previous *in vitro* studies have shown the importance of NSF in serotonergic and glutaminergic systems. We have previously reported that NSF interacts with SERT and traffics it to the plasma membrane *in vitro* (Iwata et al., 2014). To the best of our knowledge, the present study is the first to show that haploinsufficiency of *Nsf* leads to a significant decrease in the membrane expression of SERT *in vivo*. *Nsf* also interacts with GluA2 and regulates the surface expression of GluA2-containing AMPA receptors in hippocampal neurons (Nishimune et al., 1998; Noel et al., 1999). In the present study, our results showed that membrane GluA2 expression does not change in the hippocampus of *Nsf* ^+/-^ mice. This may be due to haplo-insufficiency of *Nsf* in our mice (that is, not homozygous KO mice) and/or the whole membrane fraction was corrected instead of correcting the synaptic membrane where *Nsf* is enriched (Walsh and Kuruc, 1992). Indeed, when we measured AMPA receptor levels (GluA1-3) in the postsynaptic membrane using the FRIL technique, a significant reduction in the labeling density for synaptic GluA1-3 receptors was observed in *Nsf* ^+/-^ mice compared with *Nsf* ^+/+^ mice (Figure 6E). With this technique, we could not measure GluA2 specifically because there was no appropriate anti-GluA2 antibody available. In the forebrain, including the hippocampus and cerebral neocortex, the predominantly expressed subunits are GluA1 and GluA2, and the major neuronal population expresses AMPA receptors primarily composed of heterotetramers of GluA1 and GluA2 (Isaac et al., 2007), and NSF does not interact with GluA1 (Song et al., 1998). Therefore, a decrease in the synaptic GluA1-3 receptor density may reflect a reduction in GluA2 levels in the postsynaptic membrane. In ASD patients, partial loss of *NSF* transcription and reduced SERT and GluA2 expression at the membrane surface without a decrease in their expression at the mRNA level have been reported (Purcell et al., 2001a; Nakamura et al., 2010; Iwata et al., 2014). The *Nsf* ^+/-^ mouse is a unique model that recapitulates the molecular abnormalities observed in ASD patients.

### Haploinsufficiency of *Nsf* Leads to Autism Spectrum Disorder-like Abnormal Behaviors

Notably, most behavioral alterations in *Nsf* ^+/-^ mice we report here are relevant to core ASD symptoms. While SERT and GluA2 are implicated in the pathology of ASD, behavioral abnormalities of SERT or GluA2 homozygous KO mice are inconsistent with ASD-like behaviors. SERT homozygous KO mice show impaired locomotor function, increased anxiety, and reduced aggression and depression-like behaviors (Holmes et al., 2002; Wellman et al., 2007; Murphy et al., 2008; Bartolomucci et al., 2010). GluA2 homozygous KO mice show decreased object exploration, rearing, grooming, locomotion in a novel environment, and abnormal motor coordination and learning (Jia et al., 1996; Gerlai et al., 1998). Notably, both SERT and GluA2 heterozygous KO mice appeared to be normal (Gerlai et al., 1998; Bartolomucci et al., 2010). Interestingly, *Pten* ^+/-^ mice show impaired social interactions, and this phenotype is exacerbated by crossing with SERT^+/−^ mice (Page et al., 2009). Moreover, tryptophan depletion has been shown to exacerbate repetitive behavior and elevate anxiety in adults with autism (McDougle et al., 1996). These reports suggest that a decrease in SERT expression could increase vulnerability to ASD-like behaviors. It is possible that a modest decrease in membrane expression of SERT (48% of control) and AMPA receptors (54% of control) due to haploinsufficiency of *Nsf* may lead to ASD-like behaviors in a combined manner. Alternatively, NSF has been reported to interact with β2 adrenergic receptors and GABA_A_ receptors and is thought to affect their trafficking patterns or recycling (Cong et al., 2001; Kittler et al., 2001; Leil et al., 2004). Therefore, it is possible that membrane expression of these receptors might be altered in *Nsf* ^+/-^ mice, which may affect mouse behavior. The mechanisms underlying the behavioral abnormalities in *Nsf* ^+/-^ mice will be a subject for future investigation.

### Abnormal Postsynaptic Density in *Nsf* ^+/+^ Mice

PSD is an electron-dense structure beneath the postsynaptic membrane of excitatory synapses and is usually located at the dendritic spine tip. PSD is composed of proteins, including neurotransmitter receptors, cell adhesion molecules, scaffold proteins, signaling enzymes, cytoskeleton proteins, and membrane trafficking proteins (Sheng and Hoogenraad, 2007; Kaizuka and Takumi, 2018). Many of the glutamate receptor proteins, including AMPA receptors, are concentrated in the PSD (Dosemeci et al., 2016). PSD protein mutations, including AMPA receptors, are associated with neurodevelopmental disorders, such as ASD and schizophrenia (Coley and Gao, 2018; Kaizuka and Takumi, 2018). In the current study, *Nsf* ^+/-^ mice exhibited decreased PSD areas. One explanation for this reduction is decreased AMPA receptor levels in the IMP in *Nsf* ^+/-^ mice. However, there is a possibility that the membrane expression of other proteins localized at the PSD also decreases. Further investigation is needed to assess the reasons for the reduction in PSD areas in *Nsf* ^+/+^ mice.

### Long-Term Depression Impairment in *Nsf* ^+/-^ Mice

Synaptic AMPA receptors have been suggested to be important for synaptic plasticity, such as LTP and LTD (Luscher et al., 2000; Carroll et al., 2001; Sheng and Lee, 2001; Malenka, 2003). A previous study revealed that blockade of the NSF–GluA2 interaction by a specific peptide introduced into neurons prevented homosynaptic LTD in the hippocampal CA1 region (Luthi et al., 1999). In contrast, another study reported that AP2, a clathrin adaptor, binds to GluA2 with the same binding site as with NSF and that AP2-GluA2 binding is essential for hippocampal LTD, while NSF function is needed to maintain synaptic AMPA receptor responses but is not directly required for LTD (Lee et al., 2002). Here, we showed that LTD was impaired in the hippocampal CA1 region in *Nsf* ^+/-^ mice. Our results support those of a previous study suggesting that the interaction between NSF and GluA2 is important for LTD expression (Luthi et al., 1999). LTD dysregulation has been observed in several mouse models of ASD (Piochon et al., 2016). In the hippocampus, enhanced LTD has been reported in Fmr1^−/−^ mice (Huber et al., 2002) and Mecp2^−/−^ mice (Asaka et al., 2006), and reduced LTD has been reported in *Tsc2* ^+/−^ (Auerbach et al., 2011) and Syngap^+/−^ mice (Barnes et al., 2015). To date, the direct link between LTD deregulation and phenotypes has not been clarified in ASD patients. However, LTD-like processes are involved in synaptic pruning; therefore, LTD dysregulation in ASD may primarily manifest as deficits in developmental synaptic pruning and the optimization of connectivity in the brain (Hansel, 2019).

## Conclusion

**FIGURE 9:**
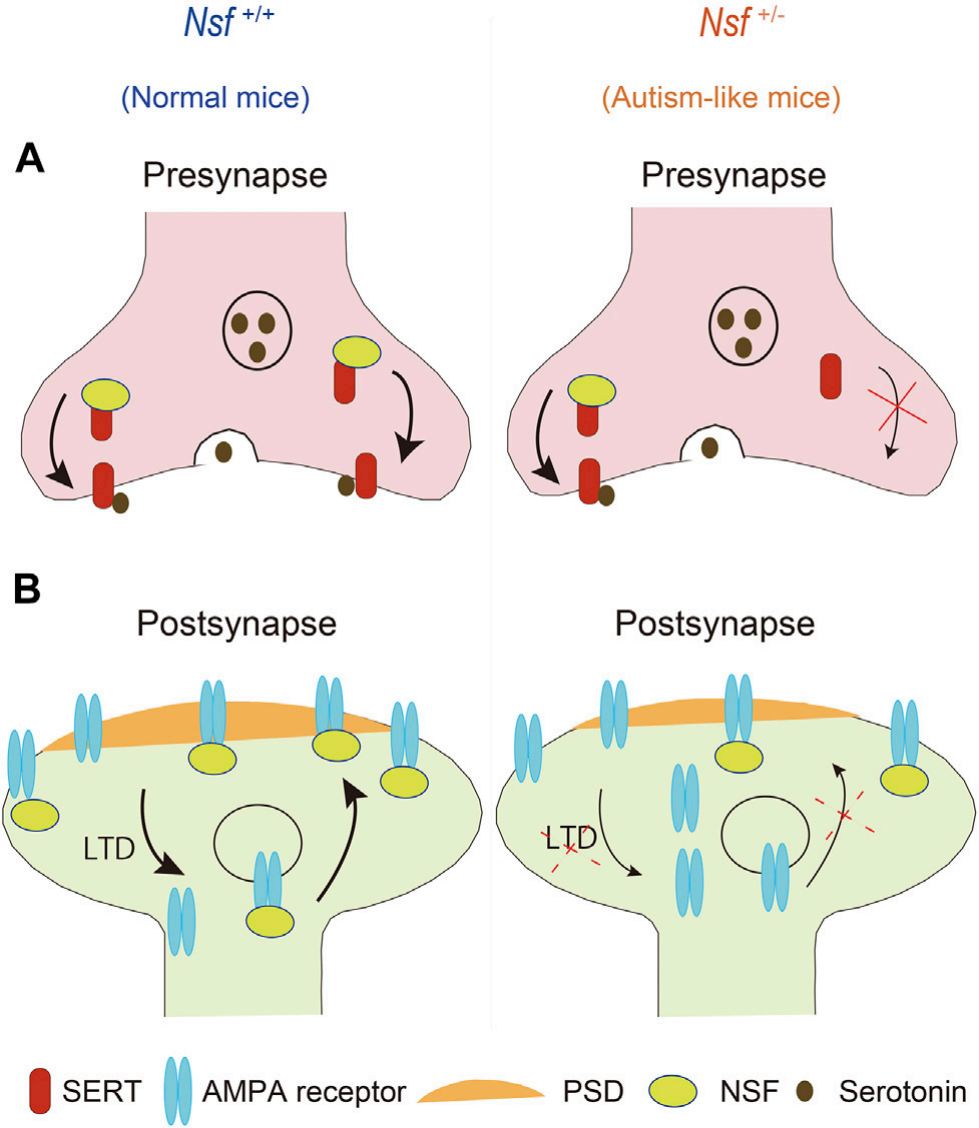
Schematic representation of possible mechanisms through which haploinsufficiency of Nsf causes neurophysiological and behavioral phenotypes similar to autism spectrum disorder via defective membrane trafficking of serotonin transporter (SERT) and alpha-amino-3-hydroxy-5-methyl-4-isoxazole propionic acid (AMPA) receptor in mice.

This study revealed that defective membrane trafficking of SERT and GluA2 due to haploinsufficiency of *Nsf* causes neurophysiological and behavioral phenotypes similar to ASD in mice (Figure 9). Although ASD showed abnormal membrane expression of many neurotransmitter receptors and transporters, including SERT and AMPA receptors, the involvement of these receptors and transporters in ASD has never been investigated simultaneously. The idea that these transmitter abnormalities have an upstream cause has not yet been discussed. To the best of our knowledge, the present study is the first to demonstrate that haploinsufficiency of *Nsf* leads to defects in the membrane expression of both SERT and AMPA receptors and causes ASD-like behavioral deficits. Notably, haploinsufficiency of *Nsf* was sufficient to develop abnormalities similar to ASD phenotypes in mice. This suggests that there may be a type of ASD with neurotransmitter and behavioral abnormalities whose root cause is the downregulation of NSF expression. Additionally, *Nsf* ^+/-^ mice provide new opportunities to explore ASD pathophysiology as a model that has neurotransmitter, neurophysiological, and behavioral phenotypes similar to ASD.

## Data Availability

The original contributions presented in the study are included in the article/Supplementary Material, further inquiries can be directed to the corresponding author.
