# Supplementary figures and images for: Autistic-Like Behavior and Impairment of Serotonin Transporter and AMPA Receptor Trafficking in N-Ethylmaleimide Sensitive Factor Gene-Deficient Mice
Source: Front Genet. 2021 Oct 20;12:748627. doi: 10.3389/fgene.2021.748627 (PMC8563833; doi:10.3389/fgene.2021.748627)

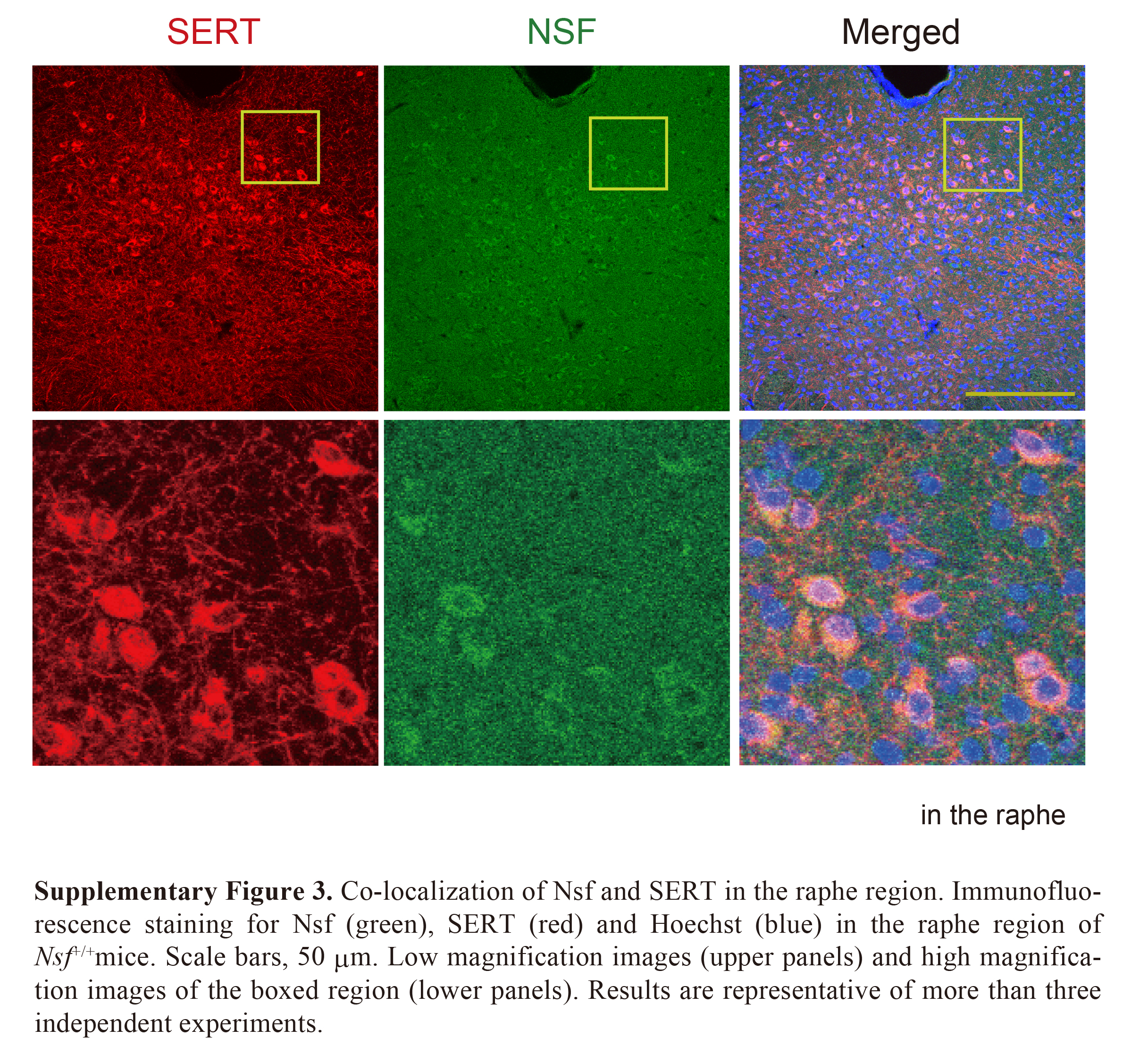

Supplement: Supplementary file 1 [file Image3.jpg]

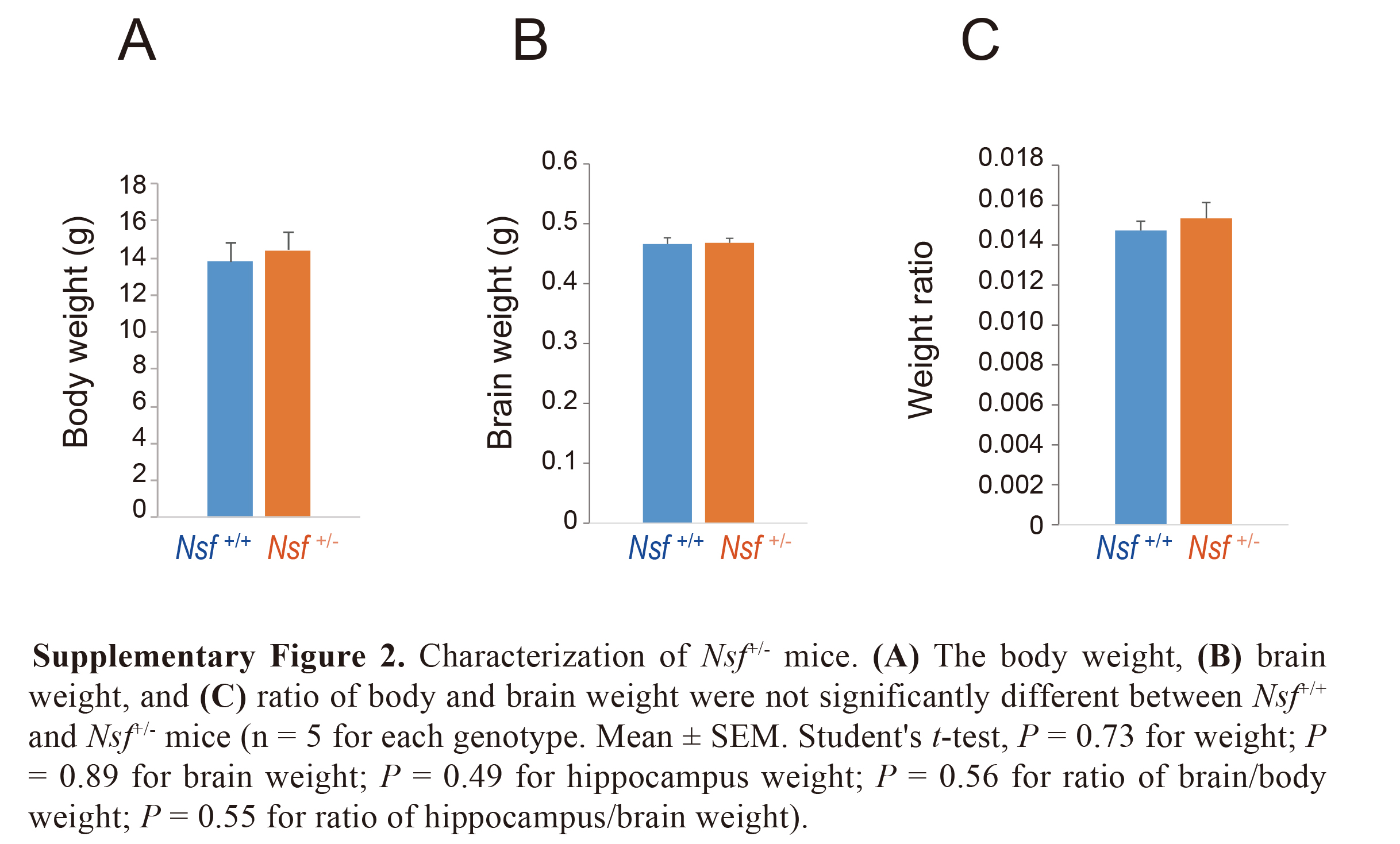

Supplement: Supplementary file 2 [file Image2.jpg]

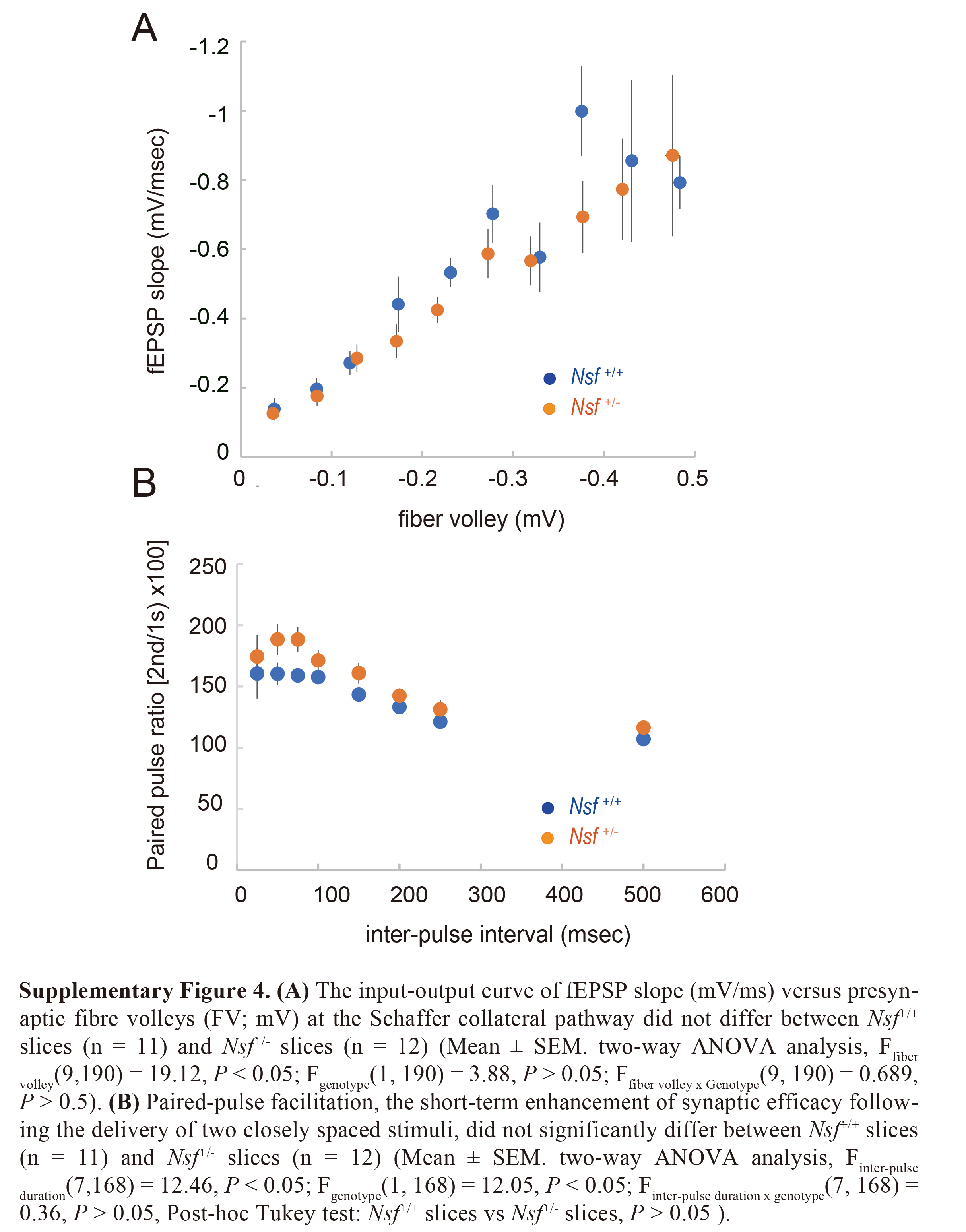

Supplement: Supplementary file 3 [file Image4.jpg]

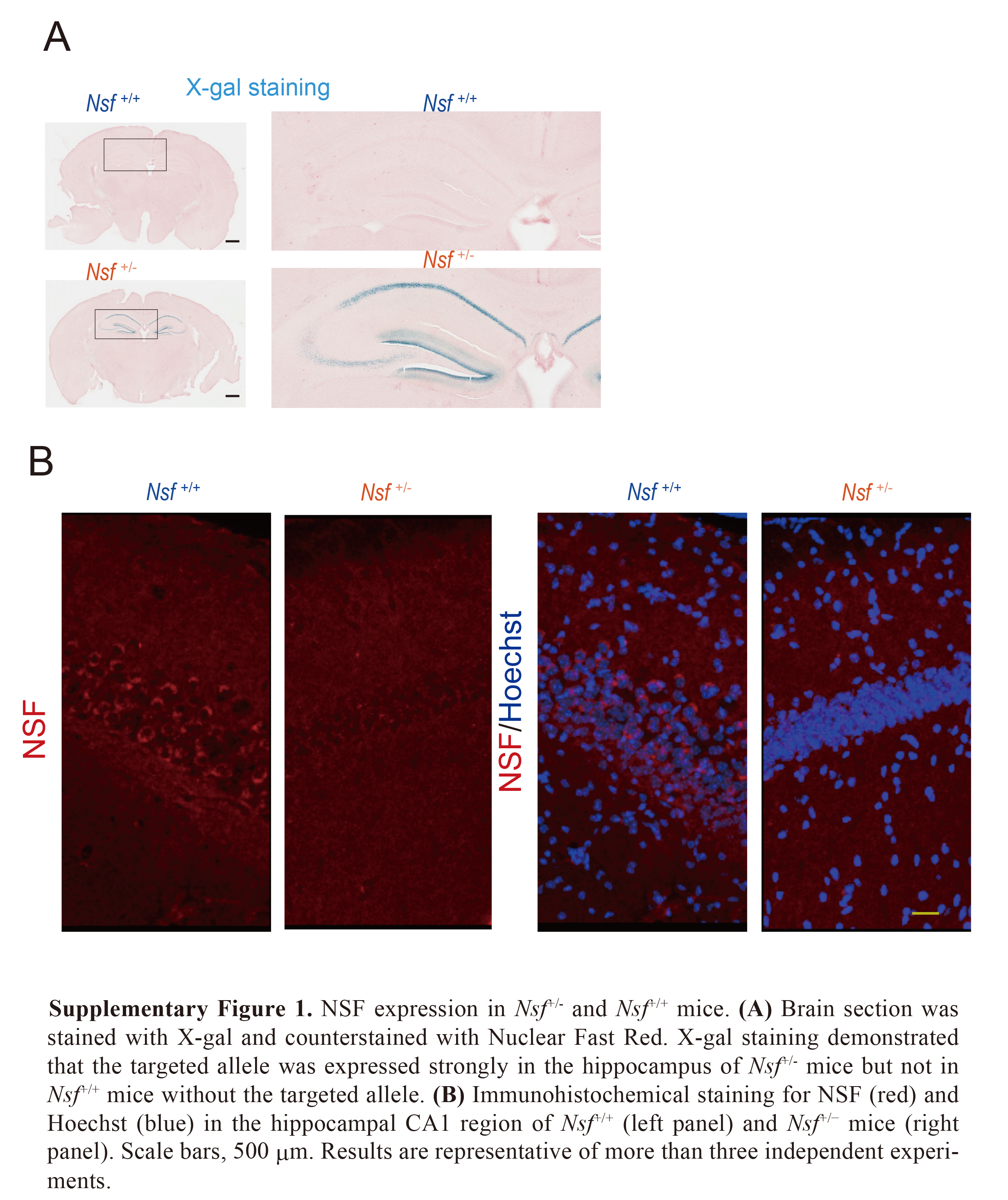

Supplement: Supplementary file 4 [file Image1.jpg]
